# Large language model for interpreting the Paris classification of colorectal polyps

**DOI:** 10.1055/a-2703-0209

**Published:** 2025-10-09

**Authors:** Davide Massimi, Luca Carlini, Yuichi Mori, Luca Di Stefano, Giulio Antonelli, Tommy Rizkala, Marco Spadaccini, Roberto de Sire, Ludovico Alfarone, Chiara Lena, Alessandro D'Aprano, Sravanthi Parasa, Raf Bisschops, Daniel von Renteln, Susanne Margaret O'Reilly, Victor Savevski, Prateek Sharma, Douglas K. Rex, Michael Bretthauer, Elena Demomi, Cesare Hassan, Alessandro Repici

**Affiliations:** 1 9268 IRCCS Humanitas Research Hospital, Rozzano, Italy; 2 18981 Department of Electronics, Information, and Bioengineering, Polytechnic University of Milan, Milan, Italy; 3 6305 Institute of Health and Society, University of Oslo, Oslo, Norway; 4 220878 Digestive Disease Center, Showa University Northern Yokohama Hospital, Yokohama, Japan; 5 638740 Ospedale dei Castelli, Ariccia, Italy; 6 Department of Gastroenterology, Swedish Medical Group, WA, United States; 7 Department of Gastroenterology and Hepatology, University Hospital Leuven, Leuven, Belgium; 8 Gastroenterology, Centre hospitalier de l'université de Montréal, Montreal, Canada; 9 Centre for Colorectal Disease, St Vincent's University Hospital, Dublin, Ireland; 10 9268 AI Center, IRCCS Humanitas Research Hospital, Rozzano, Italy; 11 Gastroenterology, University of Kansas School of Medicine and VA Medical Center, Kansas City, United States; 12 12250 Division of Gastroenterology/Hepatology, Indiana University School of Medicine, Indianapolis, United States; 13 Department of Gastroenterology, Oslo University Hospital, Rikshospitalet, Oslo, Norway; 14 9268 Endoscopy Unit, IRCCS Humanitas Research Hospital, Rozzano, Italy; 15 437807 Department of Biomedical Sciences, Humanitas University, Milan, Italy

**Keywords:** Endoscopy Lower GI Tract, Polyps / adenomas / ..., Colorectal cancer, Tissue diagnosis, CRC screening, Diagnosis and imaging (inc chromoendoscopy, NBI, iSCAN, FICE, CLE...)

## Abstract

**Background and study aims:**

Reporting of colorectal polyp morphology using the Paris classification is often inaccurate. Multimodal large language models (M-LLMs) may support morphological assessment. This study aimed to evaluate the accuracy of an M-LLM (GPT-4o) in classifying colorectal polyp morphology compared with expert and non-expert endoscopists.

**Patients and methods:**

We used the SUN dataset of colonoscopy videos from 100 unique colorectal polyps, each labeled with the validated Paris classification. An M-LLM (GPT-4o) classified five representative frames per lesion. Three expert and three non-expert endoscopists, blinded to one another, performed the same task. The primary outcome was accuracy in differentiating non-polypoid (IIa/IIc) from polypoid (Is/Ip/Isp) lesions. The secondary outcome was accuracy in differentiating sessile (Is) from pedunculated (Ip/Isp) lesions. Given the exploratory design, no multiplicity correction was applied; point estimates are presented with 95% confidence intervals (CIs), and *P* values are interpreted descriptively.

**Results:**

M-LLM accuracy for differentiating non-polypoid from polypoid lesions was 73% (95% CI 63%-81%), comparable to experts (75%, 65%-83%; *P* = 0.84) and non-experts (77%, 68%-85%; *P* = 0.52), with similar sensitivity and specificity. Accuracy for differentiating sessile from pedunculated lesions was 55% (95% CI 42%-67%), lower than experts (76%; *P* = 0.02) and non-experts (77%; *P* = 0.01), primarily due to poor specificity (12% vs. experts 82% and non-experts 88%; *P* < 0.01 for both comparisons).

**Conclusions:**

M-LLMs performed comparably to endoscopists in distinguishing non-polypoid from polypoid lesions but failed to reliably identify pedunculated morphology.

## Introduction

Use of the Paris classification is encouraged because it provides a clinically relevant morphological definition of colorectal neoplasia 1 . However, significant variability has been reported among expert endoscopists applying this classification, resulting in poor interobserver agreement, particularly with non-polypoid lesions 2 . This inconsistency could impact clinical decision-making.

Artificial intelligence (AI), particularly deep learning models, has emerged as a potential solution to reduce interobserver variability for both polyp detection and histology prediction 3 4 5 . Some models have shown promise in classifying colorectal polyp morphology according to the Paris system, reaching high accuracy and improving recognition of flat or depressed polyps, which are often misclassified by endoscopists 6 . Nonetheless, deep learning methods have limitations, primarily due to reliance on extensive supervised training datasets requiring resource-intensive manual annotations. These factors restrict dataset size and diversity, increase costs, and hinder broad clinical adoption.

Recently, use of multimodal large language models (M-LLMs) is attracting considerable attention for image interpretation, although LLMs were originally developed for text interpretation and creation. Based primarily on unsupervised learning from extensive general knowledge sources 7 , M-LLMs eliminate the need for costly image annotations and offer enhanced semantic contextualization capabilities. This potentially facilitates clinical decisions by interpreting medical images endoscopy.

We previously demonstrated that M-LLMs show moderate-to-high accuracy in colorectal polyp detection, comparable to commercially available deep learning-based computer-aided detection systems 8 . The present study aimed to assess M-LLM performance in classifying colorectal polyps according to the Paris classification.

## Methods

### Study design

The study was conducted on colonoscopy videos from SUN database (SUN Showa University Northern Yokohama Hospital, Japan), comprising 99 Japanese patients with 100 unique polyps 9 . All polyps were registered with the Paris classification and final histology. Paris classification of each polyp was labelled by endoscopists who performed the colonoscopy. These endoscopists were either experts or non-experts working under the supervision of experts, who verified the Paris classifications of the polyps. Furthermore, the classifications were double-checked later by three external experts who had performed more than 10,000 colonoscopies. The SUN database is not freely available and not publicized on the internet because its use requires a written agreement with the data provider. This restriction limits its applicability for training existing M-LLMs. Details about the SUN database are presented in Table 1 .

**Table:** **Table 1** SUN database characteristics.

SUN Showa University Northern Yokohama Hospital, Japan	
Number of patients (n)	99 Japanese patients
Number of polyps (n)	100
Median polyp size (IQR)	5 mm (3–7 mm; min: 2 mm; max 18 mm)
Diminutive polyps (≤ 5 mm)	60
**Polyp histology n (%)**	
Adenoma	87 (87%)
Non-adenoma	13 (13%)
Polypoid/non-polypoid ratio	66/34
Paris classification distribution	Is: 49, IIa: 34, Isp: 9, Ip: 8
Protruded lesions breakdown	Pedunculated (Ip/Isp): 17, sessile: 49
Endoscopy imaging modality	White-light, high-definition (HD)
IQR, interquartile range.	

### Multimodal large language model assessment

To enable analysis by M-LLMs, videos of all 100 polyps were segmented into 1,364 frames. An expert endoscopist (who was not involved in the Paris classification assessment) selected the five most representative frames for each unique polyp’s video and then submitted, via locally hosted GPT API, to OpenAI's GPT-4o (version: gpt-4o-2024–05–13; GPT) 10 . The prompt used for M-LLM analysis of the selected frames comprised a first row that was simply a clarification of the required task and the second row presented an explicit request to clarify each step of the classification reasoning to use the performance improvements offered by chain of thought. Details on the prompt analysis are reported in **Appendix 1** and **Supplementary Fig. 1** . GPT-4o was set up not to use the uploaded information for further learning.

**Fig. 1:**
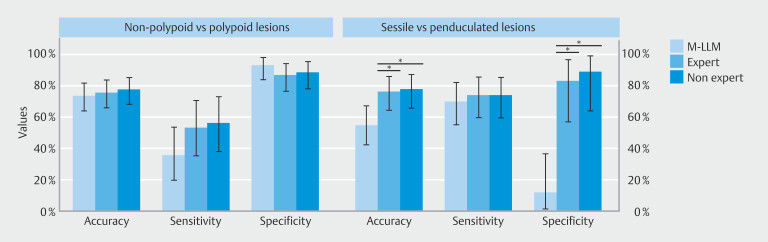
Bar plot of outcomes summary results.

### Generative AI to extract LLM representation

To further understand the knowledge of GPT-4o regarding the Paris classification, we tested its ability to generate images corresponding to specific polyp morphologies. A prompt (Appendix 1) was used to generate three polyp per Paris class in the dataset via LLMs. Three expert endoscopists visually compared these images with corresponding SUN images to identify potential reasons for accuracy pitfalls.

### Endoscopist assessment

Each lesion's video frames were independently assessed by three expert and three non-expert endoscopists, who recorded their diagnoses using the Paris classification criteria. Endoscopists who had performed at least 1,000 colonoscopies were defined as experts.

### Outcomes

The primary outcome was accuracy with which the M-LLM, relative to expert and non-expert endoscopists, distinguished non-polypoid lesions (Paris IIa and IIc) from polypoid—or protruded—lesions (Paris Is, Ip and Isp). Secondary outcomes comprised: 1) corresponding sensitivity and specificity of the M-LLM versus experts and non-experts for this same dichotomous task; and 2) interobserver agreement, expressed as Fleiss’ κ and mean pair-wise percentage agreement, calculated separately for the three experts, the three non-experts, and three independent context-reset runs of the M-LLM.

Performance, therefore, was examined for two clinically relevant comparisons. First, we assessed how accurately each rater group differentiated non-polypoid from polypoid lesions as defined above. Second, within the subset of protruded lesions, we evaluated their ability to distinguish sessile morphology (Is) from pedunculated morphology (Ip and Isp). Interobserver agreement was specifically analyzed for the M-LLM because its output is known to vary over time, necessitating an assessment of internal consistency.

### Statistical analysis

Accuracy, sensitivity, and specificity of the M-LLM were compared with those of human raters using McNemar’s test, applying a continuity correction when any cell count was zero. Statistical significance was set at *P* < 0.05. Exact 95% confidence intervals (CIs) were calculated with the Clopper-Pearson method. Interobserver agreement was quantified with Fleiss’ κ and mean pair-wise percentage agreement. Point estimates are reported with their 95% CIs; *P* values are interpreted descriptively and do not imply equivalence when > 0.05.

## Results

The SUN database included colonoscopy videos of 100 small polyps. Median polyp size was determined to be 5 mm (3–7 mm), with 60 diminutive polyps (≤ 5 mm) and a polypoid/non-polypoid ratio of 66/34. The Paris classification distribution of classes was 49 Is, 34 IIa, nine Isp, and eight Ip. In detail, among protruded lesions, 17 were pedunculated (Ip/Isp) and 49 sessile.

### Non-polypoid versus polypoid lesions

**Fig. 2:**
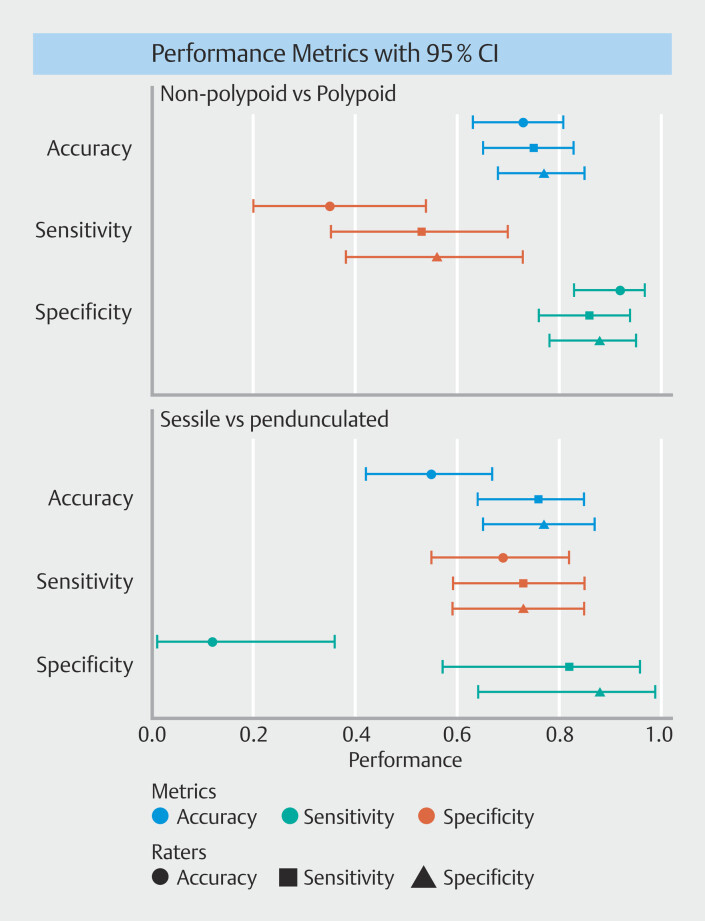
Forest plot of diagnostic performance.

For differentiation between non-polypoid (Paris IIa, IIc) and polypoid lesions (Paris Is, Ip, Isp), M‑LLM accuracy was 73% (95% CI 63%-81%) with a sensitivity and specificity of 35% (95% CI 20%-54%) and 92% (95% CI 83%-97%), respectively ( Table 2 and Fig. 1 ). Fig. 2 summarizes accuracy, sensitivity, and specificity for all rater groups with their 95% CIs. Descriptive McNemar *P* values were 0.84 for accuracy, 0.18 for sensitivity, and 0.34 for specificity versus experts, and 0.52, 0.12, and 0.45, respectively, versus non-experts. Of 22 non-polypoid lesions misclassified by M-LLM as polypoid, 20 were classified as Is and two as Ip. Whereas five polypoid lesions were misclassified as non-polypoid, four were classified as IIa and one as IIc ( **Supplementary Fig. 2** ).

**Table:** **Table 2** Performance of M-LLM in differentiating non-polypoid from polypoid lesions and sessile from pedunculated lesions.

**Non-polypoid (IIa,IIc) vs polypoid (Is,Ip,Isp) lesions**					
**Metric**	**M-LLM**	**Expert**	**Non-expert**	***P* value M-LLM vs expert **	***P* value M-LLM vs non-expert **
Accuracy (95% CI)	73% (63%-81%)	75% (65%-83%)	77% (68%-85%)	0.84	0.52
Sensitivity (95% CI)	35% (20%-54%)	53% (35%-70%)	56% (38%-73%)	0.18	0.12
Specificity (95% CI)	92% (83%-97%)	86% (76%-94%)	88% (78%-95%)	0.34	0.45
**Sessile (Is) vs pedunculated (Ip,Isp) (within polypoid lesions)**					
**Metric**	**M-LLM**	**Expert**	**Non-expert**	***P* value M-LLM vs expert **	***P* value M-LLM vs non-expert **
Accuracy (95% CI)	55% (42%-67%)	76% (64%-85%)	77% (65%-87%)	0.02	0.01
Sensitivity (95% CI)	69% (55%-82%)	73% (59%-85%)	73% (59%-85%)	0.81	0.81
Specificity (95% CI)	12% (1%-36%)	82% (57%-96%)	88% (64%-99%)	< 0.01	< 0.01
CI, confidence interval; M-LLM, multimodal large language model.					

Expert endoscopists had a sensitivity of 53% (95% CI 35%-70%), specificity of 86% (95% CI 76%-94%), and accuracy of 75% (95% CI 65%-83%), whereas non‑expert endoscopists achieved a sensitivity of 56% (95% CI 38%-73%), specificity of 88% (95% CI 78%-95%), and accuracy of 77% (95% CI 68%-85%).

### Sessile vs pedunculated lesion (within polypoid lesions)

For differentiating sessile lesions (Paris Is) from pedunculated lesions (Paris Ip, Isp), M‑LLM accuracy was 55% (95% CI 42%-67%) with a sensitivity and specificity of 69% (95% CI 55%-82%) and 12% (95% CI 1%-36%), respectively ( Table 2 and Fig. 1 ). Fig. 2 also displays estimates for sessile versus pedunculated lesions. Descriptive McNemar *P* values were 0.02 for accuracy and < 0.01 for specificity versus experts, and 0.01 and < 0.01 versus non-experts. Of 17 pedunculated polyps, 15 were misclassified as Is ( **Supplementary Fig. 2** ).

Accuracy, sensitivity, and specificity of expert endoscopists was 76% (95% CI 64%-85%), 73% (95% CI 59%-85%), and 82% (95% CI 57%-96%), respectively, whereas corresponding values for non‑expert endoscopists were 77% (95% CI 65%-87%), 73% (95% CI 59%-85%), and 88% (95% CI 64%-99%), respectively.

Accuracy, sensitivity, and specificity values for each rater for individual classes of Paris classification are provided **in Supplementary Table 1** . Paris classification results based on the SUN database, M-LLM, expert, and non-expert of each polyp are reported in **Appendix 2** . Confusion matrix results are reported in **Supplementary Fig. 3** .

### Interobserver agreement of M-LLMs and endoscopists

Agreement varied across rater groups ( **Supplementary Table 2** ). Expert endoscopists showed moderate concordance, with Fleiss κ = 0.500 and a mean pair-wise agreement of 0.673. Non-experts were slightly lower (κ = 0.462; pair-wise 0.640). The three context-reset M-LLM runs yielded κ = 0.522 and a mean pair-wise agreement of 0.767.

### AI generative for explaining LLM pitfalls

**Fig. 3:**
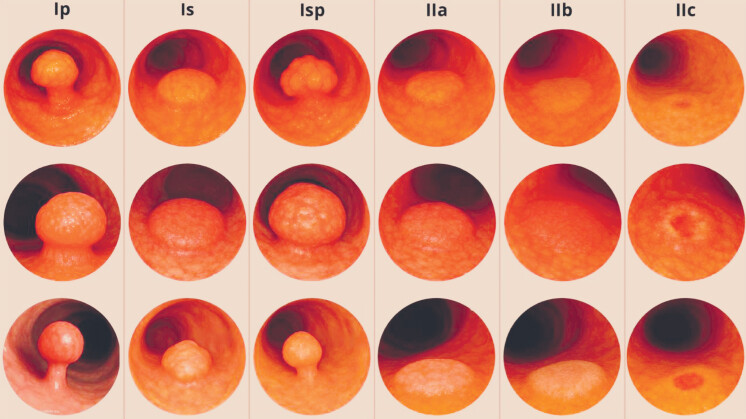
GPT-generated images of colorectal lesions classified by Paris morphology.

Generative AI representations of pedunculated lesions (Ip and Isp) revealed key anatomical inaccuracies in synthetic images. GPT-generated images depict the lesion as a geometrical spherical mass protruding from the mucosa with an unnaturally cylindric stalk. The images do not incorporate realistic physical constraints such as gravity and tissue pliability: in actual endoscopic images, stalks often bend or lay down softly along the mucosal surface due to their softness and weight. This feature is systematically absent in synthetic outputs, leading to rigid, upright depictions that do not reflect endoscopic reality. Fig. 3 shows GPT-generated colorectal lesions based on Paris classification: Three samples per each Paris class were generated and shown.

## Discussion

M-LLM showed moderate accuracy in distinguishing non-polypoid from polypoid lesions but underperformed in identifying pedunculated morphology, likely due to an oversimplified internal representation of stalks, as revealed by generative analysis. Compared with human readers, M-LLM performed similarly in classifying non-polypoid versus polypoid lesions.

All groups showed high specificity for identifying protruded polyps as polypoid, indicating reliable exclusion of protrusion when classifying lesions as non-polypoid. However, sensitivity for detecting non-polypoid lesions was low, resulting in frequent misclassification. This limitation likely stems from inherent ambiguity within the Paris classification, particularly given the predominance of small lesions (< 5 mm) in the SUN database, which complicates height-based criteria.

Misclassification of IIa lesions as sessile aligns with previous research demonstrating increased interobserver agreement when combining Is and IIa lesions 2 . In contrast, M-LLM performed poorly in differentiating pedunculated from sessile lesions. Specifically, 88% (15/17) of pedunculated lesions (classes Ip and Isp) were misclassified as sessile. Given that only 17 stalk‑bearing lesions (8 Ip and 9 Isp) were available, these point estimates carry wide 95% CIs and low specificity; consequently, all conclusions regarding pedunculated morphology remain exploratory and underscore the need for larger, balanced datasets to train and evaluate future models.

The extracted generative AI synthetic images further illustrated this deficiency, showing rigid, non-physiological stalks compared with the flexible stalk morphology present in SUN dataset images. These generated images suggest that LLMs lack an adequate internal representation of the stalk, indicating the need for targeted fine-tuning —i.e., further training the model on a curated set of accurately annotated pedunculated examples to enhance its sensitivity to subtle stalk features. Concerning interobserver agreement, M-LLM demonstrated the highest inter-rater consistency, exceeding both expert and non-expert endoscopists, values that are in line with previously reported moderate agreement among human observers for the Paris classification 2 .

M-LLMs’ good accuracy in differentiating non-polypoid morphology was a surprise, given that no object-oriented learning has been done in M-LLM. On the other hand, it may be assumed that M-LLM failure in recognizing a pedunculated morphology in the present study may be due to a limited representation of less frequent polyp types (Isp and Ip) in publicly available images and datasets on the internet on which M-LLM training was based. Performance pitfalls by M-LLMs might reflect dataset imbalance rather than inherent model limitations. Of note, in a previous study on polyp detection, we showed that M-LLM was in general moderately to highly accurate in detection of the lesion, but very poor in its segmentation. This indicates that each task of LLM in endoscopic diagnosis must be specifically validated because LLM accuracy may vary according to the individual task.

The primary limitation of our study is the inherent complexity and subtlety of Paris-based morphological distinctions, which challenge both human and AI raters. This is compounded by the modest, single-center SUN cohort (100 polyps), and therefore, external validation in larger multicenter datasets is warranted. Finally, because the study was exploratory and no multiplicity correction was applied, a risk of type-I error inflation remains. This limitation is further underscored by our expert endoscopists' low sensitivity for non-polypoid morphology, reflecting the Paris classification intrinsic uncertainty 2 . Nevertheless, this likely did not affect our outcomes, because the direct comparison between M-LLM and human endoscopists revealed divergent patterns in the two evaluated tasks, aligning M-LLM with human behavior only in one. It could be argued that comparing M-LLM directly with human diagnosis might have been more appropriate, given the uncertain reference standard; however, supplementary material analysis showed no impact on interpreting our results. Another limitation is our decision to evaluate clinically relevant scenarios based on the Paris classification instead of reporting accuracy per individual class, although supplementary data confirm this dichotomized approach's equivalence in interobserver agreement 2 . In addition, we grouped semi-pedunculated (Isp) with pedunculated (Ip) lesions to maximize the stalk‑bearing sample size; reclassifying Isp with sessile Is polyps would leave only eight true pedunculated lesions, widening the 95% CIs and rendering conclusions about pedunculated morphology purely exploratory.

Lastly, SUN images lacked biopsy forceps for estimating polyp height, but this limitation applies equally to clinical practice, affecting both M-LLM and human observers.

## Conclusions

This study underscores the potential and current limitations of M-LLMs in applying the Paris classification to colorectal polyps, notably their inability to reliably distinguish certain morphologies, especially underrepresented pedunculated lesions. However, the acceptable accuracy of M-LLMs in differentiating between polypoid and non-polypoid lesions, coupled with their capability for clinical contextualization, suggests promising applications in endoscopic assessment and reporting. Clinically, an M-LLM could run alongside CADe systems to highlight subtle non-polypoid contours in real time, write the corresponding Paris code directly into the structured endoscopy report, and post-procedure mine stored videos to populate morphology-based quality dashboards. In research, the same model may pre-label large multicenter image banks, generate photorealistic stalk-bearing polyps to balance training datasets, and serve as an interactive tutor that quizzes trainees on Paris classification. Future improvements require targeted fine-tuning of M-LLMs using balanced, domain-specific annotated datasets, emphasizing stalk morphology, to optimize their effectiveness in specialized endoscopic tasks.

## Supplementary Material

Supplementary Material Zusatzmaterial
