# Supplementary material for: Large language model for interpreting the Paris classification of colorectal polyps
Source: Endosc Int Open. 2025 Oct 9;13:a27030209. doi: 10.1055/a-2703-0209 (PMC12511921; doi:10.1055/a-2703-0209)
Supplement: Supplementary file 1 — Supplementary Material [file 10-1055-a-2703-0209_27033312.pdf]

Supplementary materials

Table of contents

Appendix 1: M-LLMs prompt analysis example report. 2

Appendix 2 Paris Classification of SUN dataset, M-LLM, expert and non-expert. 4

Supplementary Table 1 Performance of M-LLM model, expert, and non-expert assessments on the SUN dataset for the individual classes of Paris classification. . 9

Supplementary Table 2 Interobserver agreement between endoscopists and M-LLM. 10

Appendix 1: M-LLMs prompt analysis example report.

1) Prompt for M-LLMs to classify polyps according to Paris classification

The Paris classification is a widely used system for categorizing gastrointestinal lesions based on their morphology, which helps guide diagnosis, treatment, and prognosis. Example of frames used is presented in **Supplementary Figure 1**. Below is a step-by-step analysis of the lesion in the provided image to determine its Paris classification:

Step 1: **Examine the Morphology**
The lesion in the image appears as a protruding growth with a rounded, dome-like shape. It is elevated above the mucosal surface and does not appear to have a stalk (pedunculated).
- **Key observation:** The lesion is sessile (broad-based) rather than pedunculated or flat.

Step 2: **Determine the Paris Classification Type**
The Paris classification system includes the following main categories:
1. **Type 0-I:** Protruding lesions
  - **0-Ip (pedunculated):** Lesion with a stalk.
  - **0-Is (sessile):** Lesion without a stalk, broad-based.
2. **Type 0-II:** Non-protruding, superficial lesions
  - **0-IIa (slightly elevated):** Slightly raised above the mucosa.
  - **0-IIb (flat):** Flat, flush with the mucosa.
  - **0-IIc (slightly depressed):** Slightly below the mucosa.
3. **Type 0-III:** Excavated lesions (deeply depressed).
- **Assessment of the lesion in the image:** The lesion is clearly protruding but lacks a stalk, making it a **Type 0-Is (sessile)** lesion.

Step 3: **Consider Additional Features**
- **Surface characteristics:** The lesion appears smooth and uniform, without ulceration or significant irregularities.
- **Size and shape:** While size is not directly measurable from the image, the lesion's dome-like shape and sessile nature are consistent with a Type 0-Is classification.

Step 4: **Final Classification**
Based on the lesion's morphology and characteristics, the most appropriate Paris classification is:
Type 0-Is (sessile protruding lesion).

Rationale for the Classification
- The lesion is elevated above the mucosa, ruling out flat or depressed types (Type 0-II or 0-III).
- It lacks a stalk, distinguishing it from pedunculated lesions (Type 0-Ip).
- Its broad-based, dome-like appearance is characteristic of sessile lesions (Type 0-Is).
This classification is important for determining the appropriate management strategy, such as endoscopic resection or further histopathological evaluation.

2) Generative AI prompt to extract M-LLM representation

As the OpenAI API did not support image generation with GPT-4o at the time of this study, we conducted our experiments using the ChatGPT interface with the GPT-4o model. This choice does not compromise the reliability of the results, as the underlying model is the same or differs only by minor updates. Produced images are reported here without any post processing.

The prompt used was: *"For research and educational purposes, generate a medically-styled synthetic endoscopic image that simulates a colorectal lesion with characteristics consistent with the Paris classification type Ip. The image should have realistic lighting, color tones, and textures typically seen in high-resolution colonoscopic imaging, while being clearly labeled as synthetic and not derived from real patient data."*

Endosc Int Open | DOI 10.1055/a-2703-0209 | © The Author(s).

Appendix 2 Paris Classification of SUN dataset, M-LLM, expert and non-expert.

	Sun Dataset	Expert 1	Expert 2	Expert 3	Non-expert 1	Non-expert 2	Non-expert 3	M-LLM 0	M-LLM 1	M-LLM 2
0	Is	Is	Is	Is	Is	Is	Is	Is	Is	Is
1	Is	Is	Is	IIa	Is	Is	IIb	Is	Is	Is
2	IIa	IIb	IIa	IIa	IIa	IIb	IIa	IIa	IIa	IIa
3	Is	Is	Is	Is	Is	Is	Is	Is	Is	Is
4	IIa	Is	Is	IIb	Is	Is	IIb	Is	Is	IIa
5	IIa	IIa	IIa	IIa	IIa	IIa	IIa	IIa	IIa	IIa
6	IIa	Is	Isp	Is	Is	Is	Is	Is	Is	Is
7	Isp	Is	IIa	Is	Is	Is	IIa	Is	Is	Is
8	Is	Is	IIa	Is	Isp	Isp	Isp	Is	Is	Is
9	IIa	IIa	IIa	IIa	IIa	IIa	IIa	Is	IIa	Is
10	IIa	IIa	IIa	IIa	Is	Is	Is	Is	Is	Is
11	Is	IIa	Is	Is	Is	Is	Is	Ip	Ip	Ip
12	Is	Isp	Is	Is	Is	Is	Is	Ip	Ip	Ip
13	IIa	Is	Is	IIb	IIa	IIa	IIa	IIb	IIb	IIb
14	Is	IIa	Is	Is	Is	Is	IIa	Is	Is	Is
15	Is	Is	Is	IIa	IIa	Is	Is	Ip	Ip	Is
16	Is	Isp	Is	Is	Isp	Is	Is	Is	Is	Is
17	Is	IIa	Is	IIa	Is	Is	IIa	Is	Is	Is
18	IIa	IIb	Is	IIb	IIa	IIa	IIa	IIa	IIa	IIb
19	IIa	IIa	IIb	IIa	Is	Isp	Is	Is	Is	Is

	Sun Dataset	Expert 1	Expert 2	Expert 3	Non-expert 1	Non-expert 2	Non-expert 3	M-LLM 0	M-LLM 1	M-LLM 2
20	IIa	Is	Is	IIb	IIa	IIa	Is	Is	Is	IIa
21	IIa	Is	IIa	IIa	Is	Is	Is	IIa	Is	Is
22	Ip	Is	Is	Isp	Is	Is	Is	Is	Is	Is
23	Ip	Ip	Ip	Isp	Isp	Ip	Ip	Is	Is	Is
24	Is	Isp	Isp	Isp	Is	Is	Isp	Is	Is	Is
25	Is	Isp	Isp	Isp	Is	Isp	Is	Is	Is	Is
26	Is	Is	IIa	IIa	Is	Isp	Is	Is	Is	Is
27	Is	Is	IIa	IIa	IIa	IIa	Is	IIa	IIa	IIa
28	Isp	Ip	Ip	Ip	Ip	Ip	Isp	Is	Is	Ip
29	IIa	Is	Is	Is	Is	Is	Is	Ip	Is	Ip
30	Ip	IIa	IIa	IIa	IIa	IIa	Isp	Is	Is	Is
31	Ip	Ip	Ip	Isp	Isp	Ip	Ip	Is	Is	Is
32	Is	Is	Is	Is	Is	Is	Is	IIa	IIa	IIa
33	Is	IIa	Is	Is	Is	IIa	Is	Is	Is	Is
34	Ip	Ip	Ip	Ip	Ip	Ip	Ip	Is	Is	Is
35	IIa	IIa	IIa	IIa	IIa	IIa	Is	Is	Is	IIa
36	Is	Is	IIa	IIa	Isp	Isp	Isp	Is	Is	Is
37	Is	Is	Is	IIa	Is	Is	Is	Is	Is	Is
38	IIa	IIa	IIa	Is	IIa	IIa	Is	Is	Is	IIa
39	IIa	Is	Is	IIa	Is	IIa	Is	IIa	IIa	IIa
40	IIa	IIa	Is	Is	Is	IIa	IIa	Is	Is	IIa
41	Is	Is	IIa	Is	Is	Is	IIa	Is	Is	IIa

	Sun Dataset	Expert 1	Expert 2	Expert 3	Non-expert 1	Non-expert 2	Non-expert 3	M-LLM 0	M-LLM 1	M-LLM 2
42	Isp	IIa	Is	IIa	Is	IIa	IIa	Is	Is	Is
43	IIa	Is	Is	IIa	Is	IIa	Is	IIa	IIb	IIa
44	Is	Is	Is	Is	IIa	IIa	IIa	IIa	IIa	IIa
45	IIa	IIa	Is	IIa	Is	IIa	Is	IIa	IIb	IIa
46	Is	IIa	IIa	IIa	IIa	IIa	IIa	IIa	Is	IIa
47	Is	Is	Is	Is	Is	Is	Isp	Is	Is	Is
48	IIa	IIa	Is	Is	Is	Is	Is	Is	IIa	Is
49	Ip	Ip	Ip	Ip	Ip	Ip	Ip	Is	Is	Is
50	IIa	IIa	IIa	IIa	IIa	IIa	IIa	Is	Is	Is
51	IIa	IIa	IIa	IIa	IIa	IIa	IIa	Is	Is	Is
52	Is	Is	Is	IIa	IIa	IIa	IIa	Is	Is	Is
53	Is	Is	Is	Is	Ip	Ip	Isp	Is	Is	Ip
54	Is	Is	Is	Is	Is	Is	Is	Is	Is	Is
55	Is	Is	IIa	Is	Is	Is	IIa	Is	Is	Is
56	Is	IIa	Is	Is	Is	Is	Isp	Ip	Is	Ip
57	IIa	Is	Is	Is	Is	Is	Is	Is	IIa	IIa
58	Isp	Isp	Ip	Isp	Isp	Isp	Isp	Is	Is	Is
59	IIa	IIa	Is	IIa	Is	IIa	IIa	Is	Is	IIa
60	Isp	Ip	Ip	Ip	Ip	Ip	Ip	Ip	Is	Is
61	Is	Is	Is	Is	Is	Is	IIa	Is	Is	Is
62	Is	Is	Is	Is	Is	Is	Is	Is	Is	Is
63	IIa	IIa	IIa	IIa	Is	Is	Isp	Is	Is	Is

	Sun Dataset	Expert 1	Expert 2	Expert 3	Non-expert 1	Non-expert 2	Non-expert 3	M-LLM 0	M-LLM 1	M-LLM 2
64	IIa	Is	Is	IIa	Is	Is	Is	IIa	Is	Is
65	Is	Is	Is	Is	Is	Is	IIa	Is	Is	Is
66	IIa	IIa	IIa	IIa	IIb	IIb	IIb	IIa	Is	Is
67	Is	Is	Is	Is	Is	Is	Is	Is	Is	Is
68	IIa	Is	Is	IIa	Is	Is	Is	Is	Is	Is
69	Ip	Ip	Ip	Ip	Ip	Ip	Isp	Ip	Ip	Is
70	Is	Is	Is	Is	IIa	Is	Is	Ip	Ip	Ip
71	Is	Is	Is	Is	Is	Isp	Is	Is	Is	Is
72	Is	Is	Is	Is	IIa	IIa	IIa	Is	IIa	Is
73	Isp	Isp	Isp	Isp	Isp	Isp	Is	Is	Is	Is
74	Is	Is	Is	Is	Isp	IIa	Isp	Is	Is	Is
75	Is	Is	Is	Is	Is	Is	Is	Is	Is	Is
76	Is	Is	Is	Is	Is	Is	Isp	Ip	Is	Is
77	Isp	Is	Is	Is	Isp	Isp	Isp	Is	Is	Is
78	Is	IIa	IIa	Is	IIa	IIa	IIa	Is	Is	IIa
79	Is	Isp	Isp	Isp	Isp	Isp	Isp	Is	Is	Is
80	Is	Ip	Ip	Ip	Isp	Isp	IIa	Ip	Ip	Ip
81	IIa	Is	Is	Is	Is	Is	Isp	Ip	Is	Is
82	Isp	Ip	Ip	Ip	Ip-	Ip	Isp	Is	Is	Is
83	Is	Is	Is	Is	Is	Is	Is	Is	Is	Is
84	IIa	Is	Is	Is	Is	IIa	IIa	Is	Is	Is
85	IIa	Is	IIa	Is	IIa	Is	IIa	Is	Is	Is

	Sun Dataset	Expert 1	Expert 2	Expert 3	Non-expert 1	Non-expert 2	Non-expert 3	M-LLM 0	M-LLM 1	M-LLM 2
86	Is	Is	Is	Is	IIa	Is	Is	Is	Is	IIa
87	Is	Is	Is	Is	Is	Is	Is	Is	Is	Is
88	Ip	Ip	Ip	Ip	Ip	Ip	Isp	Is	Is	Ip
89	Is	IIa	Is	IIa	Is	Is	Is	Is	Is	Is
90	IIa	IIa	IIa	IIa	IIa	IIa	IIa	Is	Is	IIa
91	Is	Isp	IIa	Isp	IIa	Is	Is	Ip	Is	Ip
92	Is	Isp	Isp	Isp	Isp	Isp	Isp	Ip	Ip	Ip
93	Is	Is	Is	Is	Is	Is	Is	Ip	Ip	Ip
94	Isp	Isp	Is	Isp	Isp	Is	Isp	Is	Ip	Ip
95	Is	Is	Is	IIa	IIa	Is	Is	IIc	IIc	IIc
96	IIa	IIa	IIa	IIa	IIa	IIa	Is	IIa	Is	IIa
97	IIa	IIb	IIb	IIa	IIb	IIb	IIa	IIb	IIa	IIa
98	Is	IIa	Is	Is	Is	IIa	Is	Is	Is	Is
99	IIa	Is	IIa	Is	Is	Is	Is	Is	Is	Is

Supplementary Table 1 Performance of M-LLM model, expert, and non-expert assessments on the SUN dataset for the individual classes of Paris classification.

**M-LLM model on the SUN dataset**			
**Class**	**Accuracy (%)**	**Sensitivity (%)**	**Specificity (%)**
Is	50	70	31
Isp	91	0	1
Ip	80	13	86
IIa	72	29	94
IIb	98	0	98
**Expert on the SUN dataset**			
**Class**	**Accuracy (%)**	**Sensitivity (%)**	**Specificity (%)**
Is	68	73	63
Isp	89	33	95
Ip	94	75	96
IIa	73	47	86
IIb	98	0	98
**Non-expert on the SUN dataset**			
**Class**	**Accuracy (%)**	**Sensitivity (%)**	**Specificity (%)**
Is	70	73	67
Isp	89	44	93
Ip	94	75	96
IIa	88	5	87
IIb	98	0	98

Supplementary Table 2 Interobserver agreement between endoscopists and M-LLM.

Metric	Experts	Non-experts	M-LLM runs
**Fleiss κ**	0.500	0.462	0.522
**Mean pair-wise agreement**	0.673	0.640	0.767

Supplementary Fig. 1 Example of five representative colonoscopy frames used to prompt the M-LLM.

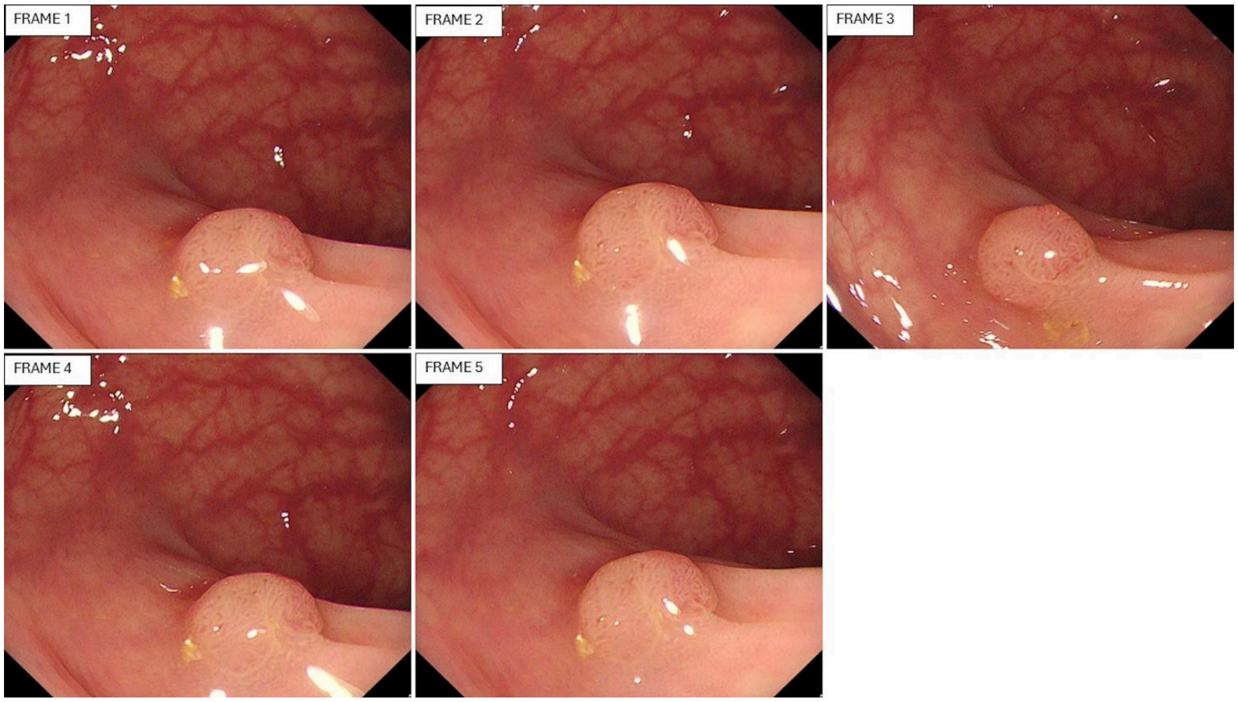

Supplementary Fig. 2 Performance errors of the multimodal-large language model (M-LLM) in polyp Paris classification tasks.

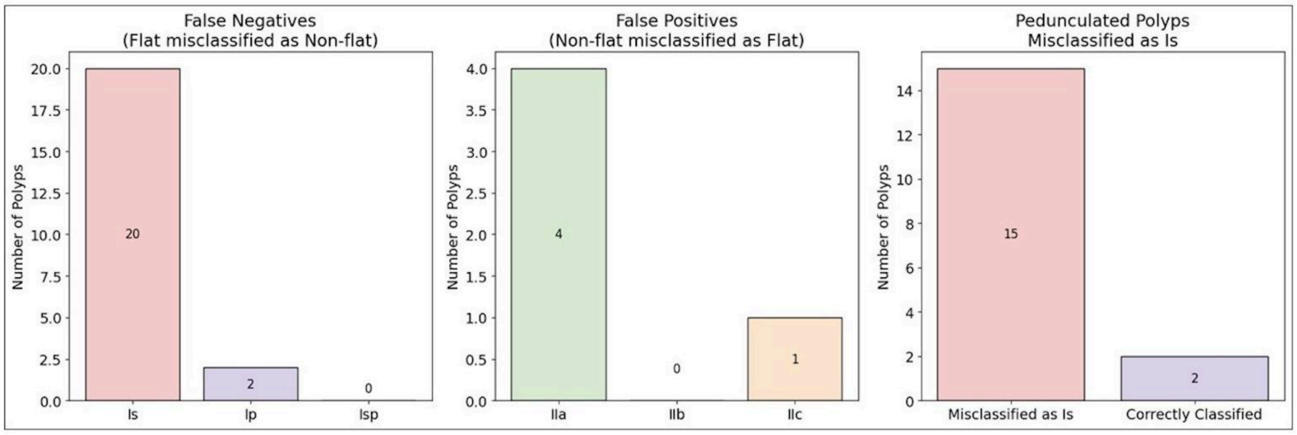

Supplementary Fig. 3 Confusion matrix.

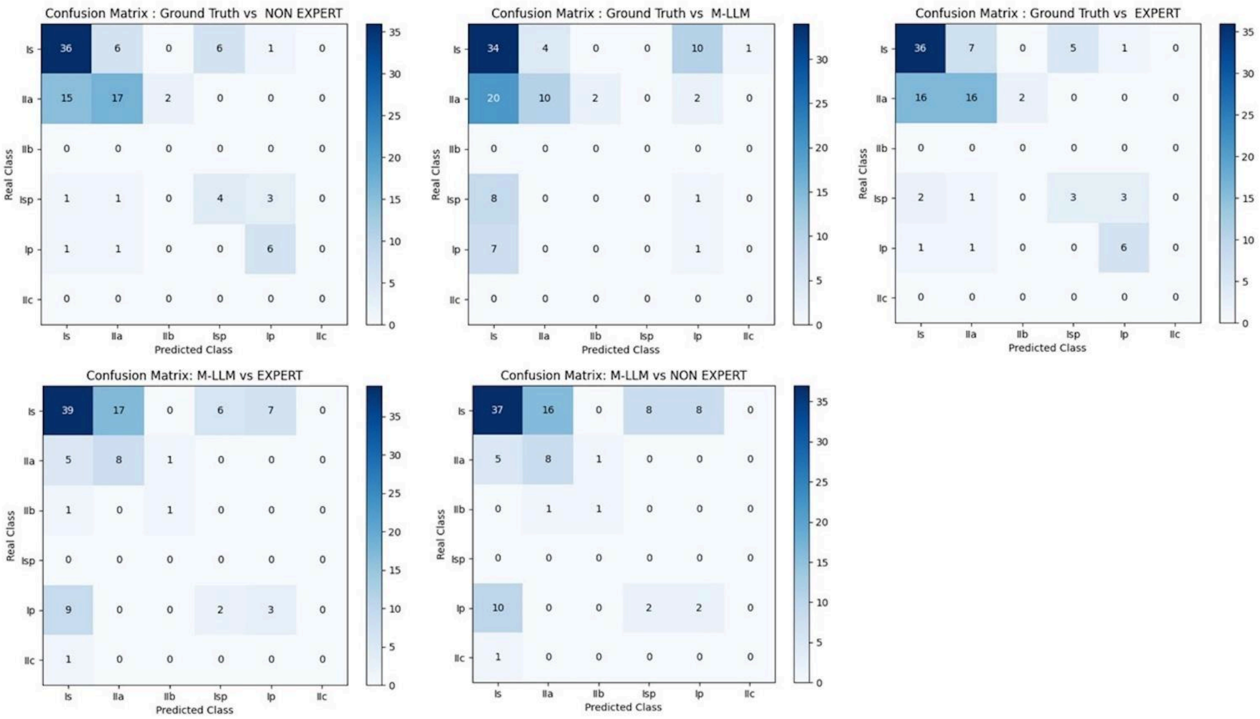

Endosc Int Open | DOI 10.1055/a-2703-0209 | © The Author(s).
